# Spectral extension and synchronization of microcombs in a single microresonator

**DOI:** 10.1038/s41467-020-19804-8

**Published:** 2020-12-14

**Authors:** Shuangyou Zhang, Jonathan M. Silver, Toby Bi, Pascal Del’Haye

**Affiliations:** 1 Max Planck Institute for the Science of Light, 91058 Erlangen, Germany; 2 National Physical Laboratory (NPL), Teddington, TW11 0LW UK; 3 City, University of London, London, EC1V 0HB UK; 4 Department of Physics, Friedrich Alexander University Erlangen-Nuremberg, 91058 Erlangen, Germany

**Keywords:** Optics and photonics, Nonlinear optics, Solitons

## Abstract

Broadband optical frequency combs are extremely versatile tools for precision spectroscopy, ultrafast ranging, as channel generators for telecom networks, and for many other metrology applications. Here, we demonstrate that the optical spectrum of a soliton microcomb generated in a microresonator can be extended by bichromatic pumping: one laser with a wavelength in the anomalous dispersion regime of the microresonator generates a bright soliton microcomb while another laser in the normal dispersion regime both compensates the thermal effect of the microresonator and generates a repetition-rate-synchronized second frequency comb. Numerical simulations agree well with experimental results and reveal that a bright optical pulse from the second pump is passively formed in the normal dispersion regime and trapped by the primary soliton. In addition, we demonstrate that a dispersive wave can be generated and influenced by cross-phase-modulation-mediated repetition-rate synchronization of the two combs. The demonstrated technique provides an alternative way to generate broadband microcombs and enables the selective enhancement of optical power in specific parts of a comb spectrum.

## Abstract (web summary)

Broadband frequency combs are a key enabling technology for frequency metrology and spectroscopy. Here, the authors demonstrate that the spectrum of a soliton microcomb can be extended by bichromatic pumping resulting in two combs that synchronize their repetition rate via cross-phase modulation.

## Introduction

Microresonator-based frequency combs (“microcombs”) provide a promising platform for miniaturizing optical frequency comb systems, and have drawn significant attention in the past decade due to their compactness, high repetition rates from 10 GHz to 1 THz, and broad spectral bandwidths^1,2^. In particular, soliton formation in microresonators has been demonstrated to generate low noise, fully coherent frequency combs^3–5^. To date, microcombs have been successfully used for optical frequency synthesis^6^, optical coherent communications^7^, laser-based light detection and ranging^8,9^, dual-comb spectroscopy^10,11^, and low noise electronic signal generation^12,13^, just to name a few.

Broadband optical frequency combs are required for precise optical frequency metrology and spectroscopy. Soliton microcombs have been demonstrated with spectral bandwidths reaching more than one octave^14–19^, and octave-spanning soliton microcombs with THz mode spacing directly realized, using Si_3_N_4_ resonators by carefully engineering their dispersion properties^14,15^. Spectra of soliton microcombs with GHz mode spacing can be externally broadened to cover an octave, using highly nonlinear fibers^16^ or nonlinear waveguides^18^. Bichromatic pumping of microresonators at similar wavelengths has been studied for microcomb generation with the benefits of a thresholdless pump intensity and stabilization of the frequency comb repetition rate^20–24^. More recently, bichromatic pumping has been demonstrated for the generation of dual orthogonally polarized microcombs both theoretically and experimentally^25,26^. Similar to bichromatic pumping, it has also been shown that a second frequency comb can be generated through Raman gain within a microresonator^27^.

Here, we demonstrate the extension of the spectral bandwidth of a soliton microcomb by bichromatic pumping. One of the pump lasers, within the C-band (1530–1565 nm), is used to generate a primary bright soliton microcomb, where the overall group velocity dispersion of the microresonator is anomalous. Another pump laser in the O-band (1260–1360 nm) is used both to compensate the thermal effect of the microresonator during soliton generation^28^ and to generate a second frequency comb via Kerr cross-phase modulation (XPM) with the primary soliton pulse, which leads to an extension of the comb spectra into the normal dispersion regime. Our results show that the two combs’ repetition rates synchronize, while maintaining an overall offset as a result of the two independent pump lasers. Numerical simulations agree well with the experimental results, and show that a bright optical pulse is passively formed and synchronized, with the primary soliton pulse via XPM. In addition, we show that a dispersive wave (DW) can be generated in the auxiliary frequency comb at a wavelength, where phase matching occurs due to the forced synchronization of the repetition rate, in contrast to conventional models, in which the DW position is determined by higher-order dispersion. These results can be used to selectively amplify the optical power in regions of microcombs that are of interest, e.g., for optical frequency metrology.

## Results

### Experimental setup for spectral extension via bichromatic pumping

**Fig. 1:**
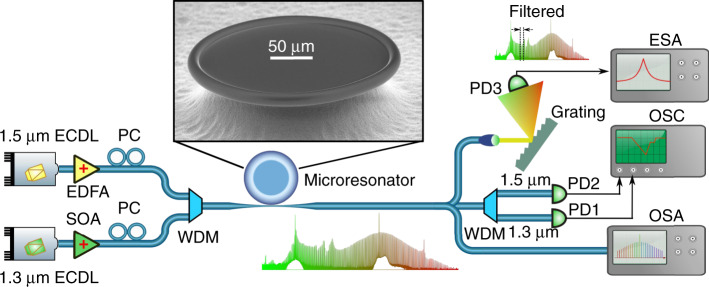
Experimental setup used for broadband microcomb generation via bichromatic pumping. The 1.5-µm primary pump laser is used to generate a soliton microcomb, while the 1.3-µm auxiliary laser thermally stabilizes the resonator and at the same time generates a second frequency comb that broadens the spectrum. ECDL external cavity diode laser; EDFA Erbium-doped fiber amplifier; SOA semiconductor optical amplifier, WDM wavelength division multiplexer; PC polarization controller; PD photodetector; OSA optical spectrum analyzer; OSC oscilloscope; ESA electronic spectrum analyzer. Inset: scanning electron microscope image of one of the 250-µm-diameter microtoroids used in the experiments.

Figure 1 shows the schematic of the experimental setup. A 1.5 μm wavelength external cavity diode laser (ECDL) with a short-term linewidth of <10 kHz is used as the first pump laser for generating a soliton microcomb, while a similar ECDL at 1.3 μm is used as a second pump laser. Here, the second pump laser plays two roles: firstly to passively stabilize the circulating optical power inside the microresonator to facilitate soliton generation from the first pump^28^, and secondly to generate a second frequency comb to extend the overall comb spectrum. For clarity, throughout this manuscript, the first pump at 1.5 μm is referred to as the primary pump and the second pump laser at 1.3 μm as the auxiliary pump. The inset in Fig. 1 shows the 250-µm-diameter fused silica microtoroid used in the experiments, with a free spectral range (FSR) of 257 GHz (ref. ^29^). The microtoroid was fabricated from a silicon wafer with a 6-µm layer of silicon dioxide^13^. A mode family of the resonator with a quality factor of ~2 × 10^8^ was chosen for the soliton generation, and the 1.5-μm primary pump laser was tuned to a wavelength around 1547.9 nm to generate the soliton microcomb. The 1.3-μm auxiliary laser is used to pump a mode of the same mode family at a wavelength of 1335 nm. The two lasers were combined with a wavelength division multiplexer (WDM) and evanescently coupled into the microresonator via a tapered optical fiber. Fiber polarization controllers were used to match the polarizations of each laser to the modes of the microresonator. At the resonator output, part of the light was monitored with an optical spectrum analyzer (OSA). Another WDM was used to separate the two pump wavelengths in order to monitor their transmissions via two photodiodes (PD1 and PD2). In addition, a reflective diffraction grating was used to spatially select part of the optical spectrum of the microcomb and to subsequently detect the filtered optical spectrum with an ultrafast photodiode (PD3, 50 GHz bandwidth).

### Spectral extension with the auxiliary mode from the primary mode family

**Fig. 2:**
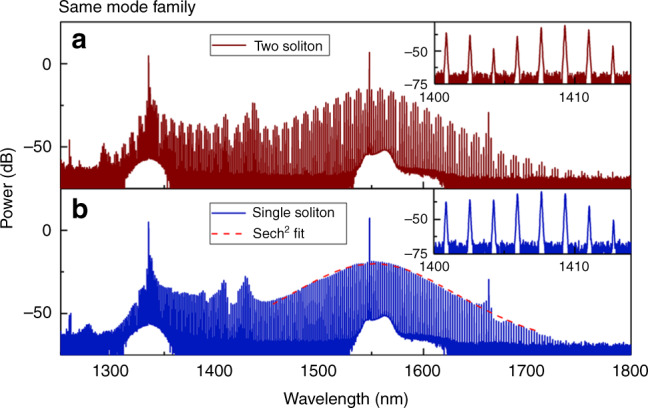
Extended optical spectra of soliton states in a 250 micron diameter microtoroid. **a** Optical spectrum with the primary microcomb in a two-soliton state. The primary pump power at 1550 nm is 250 mW, and the auxiliary laser power at 1330 nm is 150 mW. Inset: zoomed-in spectrum in the spectral range from 1400 to 1414 nm, with a resolution bandwidth of 0.02 nm. **b** Optical spectrum with the primary microcomb in a single-soliton state. The red dashed line shows a sech^2^ envelope. Inset: zoomed-in spectrum between 1400 nm and 1414 nm, with a resolution of 0.02 nm.

In the experiments, the 1.3-μm auxiliary laser was first tuned downward in frequency into its resonance, and then kept on the blue-detuned side of the resonance to passively stabilize the intracavity power and the temperature of the microresonator. The 1.5-μm primary pump laser was then tuned from the blue-detuned side of its resonance to the red-detuned soliton regime to generate a stable optical soliton. By optimizing the detuning and optical power of the 1.3-μm auxiliary laser, soliton states can be accessed by slowly tuning the primary pump laser frequency into the soliton regime^28^. In the experiments, ~250 mW of 1.5-μm primary pump light was used to generate the bright soliton microcomb, while ~150 mW of auxiliary pump light was used to passively compensate the thermally induced resonance shifts of the microresonator. Figure 2 shows typical optical spectra that are measured with the primary microcomb in a single-soliton and two-soliton state. Surprisingly, in the two-soliton state (Fig. 2a), we can see that the spectrum around the auxiliary pump wavelength (1300–1400 nm) has the same spectral envelope as the primary soliton spectrum ~1550 nm, which suggests that the constituent pulses of the auxiliary comb waveform are locked to the primary solitons in the time domain. The inset of Fig. 2a shows the region of overlap (1400–1414 nm) of the optical spectrum in between the two pump lasers. Limited by the 0.02 nm resolution of the OSA, only a single peak was resolved for each pair of comb modes in the overlapping region. Figure 2b shows the spectrum of the primary microcomb in a single-soliton state. The optical spectrum around the primary pump wavelength has a smooth, sech^2^-like envelope (red dashed line in Fig. 2b). The 3-dB bandwidth of the spectrum is ~7.4 THz, corresponding to a 50-fs optical pulse. Similarly, the inset of Fig. 2b shows the region of overlap of the single-soliton optical spectrum. Again, only one peak per FSR is observed by the OSA.

**Fig. 3:**
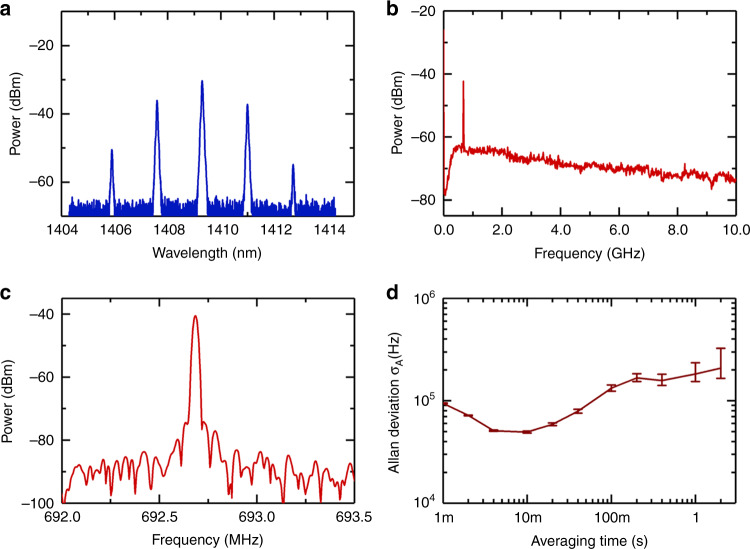
Optical spectrum in the overlapping region between the two frequency combs and corresponding RF spectra. **a** Filtered optical spectrum. **b** RF spectrum of the beat note signal between the two frequency combs from DC to 10 GHz. **c** RF spectrum of the beat signal with a 20 kHz resolution bandwidth. **d** Allan deviation of the beat note signal.

To investigate whether the frequency comb spectra in the region of overlap shown in Fig. 2 are continuous or composed of two separate frequency combs, a grating (as shown in Fig. 1) was used to spectrally filter the overlapping portion of the spectrum. When operating the primary microcomb in the single-soliton state, the filtered spectrum (covering ~5 FSR) was sent to photodetector PD3 (Fig. 3a). As shown in Fig. 3b, a single peak was observed in the radio frequency (RF) spectrum of the beat signal from DC to 10 GHz. Considering the 0.02 nm (4 GHz) resolution of the OSA, we can conclude that the optical spectra shown in Fig. 2 are composed of two different frequency combs, resulting from the bichromatic pumping. Each comb line seen on the OSA in the overlapping region actually consists of two comb lines, one from each pump. Moreover, the existence of a single peak in the RF spectrum also reveals that the comb lines generated from the auxiliary pump laser have the exact same spacing as the primary soliton microcomb. Figure 3c shows the RF spectrum of the beat signal with a resolution bandwidth of 20 kHz. The beat note has a >35 dB signal-to-noise ratio, which implies the frequency comb generated from the auxiliary laser is low-noise coherent. To characterize the relative drift between the primary soliton microcomb and the auxiliary comb, the beat signal was mixed down and measured with a frequency counter using a 1 ms gate time. Figure 3d shows the resulting Allan deviation of the beat note with fluctuations of ~180 kHz at 1 s. The offset beat note between the two combs could be locked to a RF reference for further stabilization.

### Spectral extension with the auxiliary mode from a different mode family

**Fig. 4:**
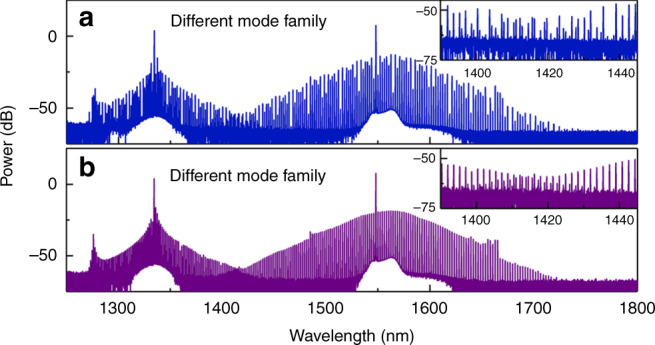
Spectral extension of microcombs across different mode families. **a** A comb spectrum with the primary soliton microcomb in a multi-soliton state. **b** A measurement with the primary soliton in a single-soliton state. The envelope pattern is transferred between the two mode families. The insets show a zoom into the overlapping spectral region of the combs.

By selecting a resonance from a different optical mode family for the auxiliary pump which does not belong to the primary pump’s mode family, similar extension of the microcomb optical spectrum can be obtained, as shown in Fig. 4a, b. Figure 4a shows the optical spectrum with the primary soliton microcomb in a multi-soliton state, when the auxiliary pump laser is operating at 1334.6 nm. Similar to the previous results, the extended spectrum around the auxiliary pump wavelength (1300–1400 nm) mimics the spectral envelope of the primary soliton, which indicates that the pulses at the auxiliary wavelength are synchronized with respect to the primary solitons. This is also supported by our numerical simulations. However, the offset between the combs in this measurement is ~90 GHz, which prevents the direct measurement of the beat note with our photodiode. Figure 4b shows the corresponding optical spectrum when the primary microcomb is in the single-soliton state.

### Numerical simulations of spectral extension

**Fig. 5:**
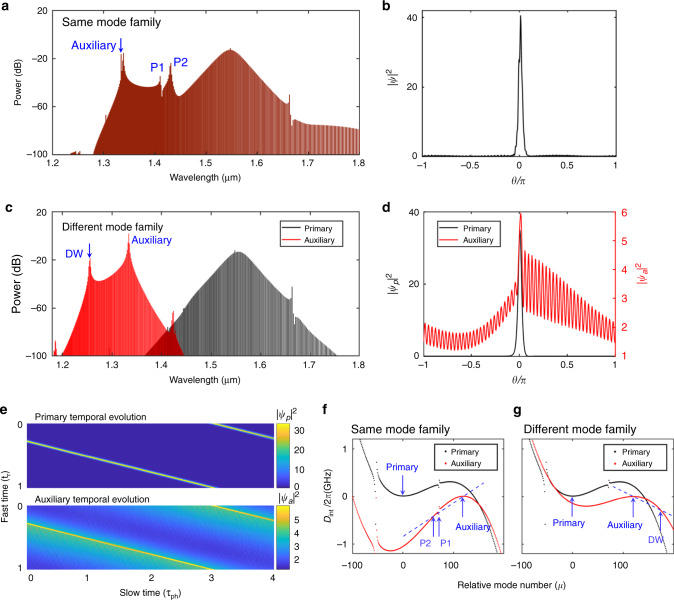
Numerical simulation of the temporal dynamics of intracavity optical fields. **a** Simulated intracavity optical spectrum with the primary comb in the single-soliton state and with the auxiliary pump mode being part of the primary pump’s mode family. **b** Temporal waveform corresponding to the spectrum shown in **a**. **c** Simulated intracavity optical spectrum with the auxiliary laser coupled into a resonance of a different mode family, which is not part of the mode family of the primary pump. DW dispersive wave. **d** Temporal waveforms (black, left axis) of the primary soliton and its induced auxiliary optical pulse (red, right axis) corresponding to the spectrum shown in **c**. **e** Temporal waveform evolution of the primary soliton (upper panel) and bright auxiliary optical pulse (lower panel) for the same parameters as in **d**. **f**, **g** Integrated dispersion *D*_int_ (*μ*) of the 250-µm-diameter silica microtoroid relative to the primary (black) and auxiliary (red) pump modes, with the auxiliary mode from the same (**f**) and different (**g**) mode family, used for the numerical simulations. The primary pump’s relative mode number is chosen to be 0, while the auxiliary pump’s relative mode number is 120. The crossing points between the dashed line and *D*_int,a_ (*μ*) in **f**, **g** show the phase-matching points between the spectral center of the auxiliary comb and the corresponding dispersive waves.

In order to gain an insight into the temporal dynamics of the intracavity primary and auxiliary fields, we performed a number of numerical simulations based on the Lugiato–Lefever equation (LLE)^30–32^, for the cases in which the auxiliary pump laser is coupled to an optical mode either belonging or not belonging to the soliton mode family. Figure 5a represents the simulated intracavity optical spectrum in the first case, when the auxiliary mode is from the soliton mode family and the primary soliton microcomb is in a single-soliton state. The simulated results are in good agreement with the experimental measurements (Fig. 2b), with two distinct peaks (DWs) in the overlapping region of the optical spectrum^33^. The simulated results are explained in more detail in the “Methods” section and in the Supplementary Information. From the simulations, we determine that the left peak, marked as P1 in Fig. 5a, is induced by an avoided mode crossing^34^, while the right one, marked as P2, is generated by the frequency-dependent dispersion of the resonator modes, where phase-matching between the auxiliary optical pulse and the DW occurs^35,36^. Note that even though the presence of higher-order dispersion does not lead to a zero crossing of the integrated dispersion^33^ of the auxiliary modes ~1430 nm (see “Methods” section), the phase-matching condition for the P2 DW still occurs as a result of XPM-forced synchronization of the repetition rate. Once stable soliton states are accessed, the repetition rate of the auxiliary optical pulse synchronizes with the primary soliton. This changes the effective local D1 coefficient for the auxiliary frequency comb, and thereby creating a phase-matching point for the P2 DW. More details about the DW generation are presented in the “Methods” section. Figure 5b is the simulated temporal waveform of a microcomb in the single-soliton state. It should be noted that the spatiotemporal modulations in the pulse pedestal are induced by the beat signals between the primary pump and the strongest auxiliary sideband, as well as the two DWs.

Figure 5c shows the simulated intracavity optical spectrum when the primary soliton microcomb is in a single-soliton state in the case, where the auxiliary laser is coupled to a mode that does not belong to the soliton mode family. These simulated results are also in good agreement with the measurements shown in Fig. 4b. Figure 5d depicts the simulated temporal waveform of a single soliton (black, left axis) generated by the primary pump laser. Surprisingly, a bright optical pulse (red, right axis) is generated in the normal dispersion regime by the auxiliary pump laser. It should be noted that the bright optical pulse is formed and trapped by the bright soliton via XPM. This indicates that the auxiliary frequency comb has the same repetition rate as the primary soliton. Figure 5e shows the corresponding evolution of the temporal waveforms. We can see that the bright auxiliary optical pulse propagates with the same group velocity as the primary soliton, which verifies the locking of the repetition rate. After the formation of the primary solitons in the microresonator, the high intensities of the primary soliton pulses modify the refractive index and create attractive potential wells for the co-propagating auxiliary pulses through XPM^37–39^. Similar to the experimentally obtained optical spectrum in Fig. 4b, there is also a peak (DW, marked with an arrow) on the blue side of the auxiliary pump in the simulated auxiliary optical spectrum, which induces the fast oscillations in the pedestal of the bright auxiliary optical pulse shown in Fig. 5d.

It should be noted that, as shown in Fig. 5c, when using an auxiliary mode that does not belong to the soliton mode family, the center frequency of the primary soliton comb envelope slightly shifts to the red side due to XPM with the auxiliary frequency comb, which is consistent with the experimental optical spectra shown in Fig. 4a, b.

## Discussion

We have demonstrated that the optical spectrum of a soliton microcomb can be extended through bichromatic pumping. The first pump laser generates a soliton microcomb in the anomalous dispersion regime, while the second laser both thermally stabilizes the microresonator and produces a second frequency comb at a repetition rate that is passively synchronized with the main soliton through cross-phase modulation. Our numerical simulations of the spectrally extended microcombs are in excellent agreement with the experimental results. Further simulations reveal the shape of the optical pulses that are generated in the normal dispersion regime via bichromatic pumping. These findings are further confirmed even when using auxiliary optical modes with different polarization to the primary comb. In addition, we report that a DW can be generated as a result of XPM between two pumps, in contrast to conventional DW generation, which is determined by the higher-order dispersion of microresonators. By measuring the offset frequency between the two combs, this technique can be used to spectrally extend frequency combs without requiring advanced dispersion engineering of the microresonators. In particular, this technique might allow bridging of spectral regions with unfavorable dispersion for broadband frequency comb generation. Moreover, the demonstrated method can be used to enhance the power of comb modes in selected spectral regions, which has applications in optical clocks and optical spectroscopy. By locking both pump frequencies to atomic frequency references, the repetition rate of the microcomb is directly related to the difference between the two pumping frequencies, and can be used to create an optical clock. We believe that the observed repetition-rate synchronization between microcombs in different spectral regions in a microresonator will be of wide interest for optical frequency metrology and spectroscopy, using microresonator-based frequency combs.

## Methods

### Numerical simulations

Figure 5f, g shows the integrated dispersion *D*_int_ profiles relative to the primary (black curve) and auxiliary (red curve) pump modes for the 250-µm-diameter silica microtoroid. In Fig. 5f, the auxiliary laser is coupled into the same mode family, and in Fig. 5g into a different mode family compared to the primary pump laser. The combined material and geometrical dispersion is given as:

1 $$D_{{\mathop{\rm{int}}} , \ast }(\mu ) = \omega _{ \ast ,\mu } - (\omega _{ \ast ,0} + D_{ \ast ,1}\mu ) = \frac{{D_{ \ast ,2}}}{{2!}}\mu ^2 + \frac{{D_{ \ast ,3}}}{{3!}}\mu ^3,$$

where *μ* is the mode number relative to either the primary or auxiliary pump mode as relevant (for which *μ* = 0), ω_*,*μ*_ is the resonance frequency of that mode, “*” refers to either the primary (“p”) or auxiliary (“a”) comb, D_*,1_/2π is the FSR of the resonator at either the primary (*D*_p,1_/2π) or auxiliary (*D*_a,1_/2π) pump mode, and D_*,2_ and D_*,3_ are coefficients of second- and third-order dispersion, respectively. From Fig. 5f, g, *D*_int,p_(*μ*) (black curve) is approximately parabolic close to the primary pump mode, indicating anomalous group velocity dispersion (*D*_p,2_ > 0), which supports the formation of bright solitons. In order to accurately simulate the experimental results shown in Figs. 2 and 4, we added avoided mode crossings around 1410 nm (*μ* = 71) and 1663 nm (*μ* = −54)^34^. In contrast, *D*_int,a_(*μ*) (red curve in Fig. 5f, g) shows normal group velocity dispersion around the auxiliary mode.

In the case, in which the auxiliary laser is coupled into an optical mode from the soliton mode family, we ran the simulations with a single generalized LLE^22^:

2 $$\frac{{\partial \psi \left( {\theta ,\tau } \right)}}{{\partial \tau }} = - (1 + i\alpha _{\mathrm{p}})\psi + i\left| \psi \right|^2\psi - \mathop {\sum}\limits_{n = 2}^{N \ge 2} {( - i)^{n + 1}\frac{{\beta _{{\mathrm{p}},n}}}{{n!}}} \frac{{\partial ^n\psi }}{{\partial \theta ^n}} + F\left( {\theta ,\tau } \right),$$

3 $$F\left( {\theta ,\tau } \right) = F_{\mathrm{p}} + F_{\mathrm{a}}\exp \left( {i\mu _{\mathrm{a}}\theta - i\left( {\alpha _{\mathrm{a}} - \alpha _{\mathrm{p}}} \right)\tau - i\frac{{2D_{{\mathop{\rm{int}}} ,{\mathrm{p}}}\left( {\mu _{\mathrm{a}}} \right)}}{{{\Delta}\omega _0}}\tau } \right),$$

where *τ* is the slow time, normalized to twice the photon lifetime (*τ*_ph_), *θ* is the azimuthal angle in a frame co-rotating at the average of the primary and auxiliary group velocities. *F*_p_ and *F*_a_ are the dimensionless external pump amplitudes, and *ψ*(*θ,τ*) is the intracavity field envelope driven by both pump terms *F*_p_ and *F*_a_. *α*_p_ and *α*_a_ are the frequency detunings from the primary and auxiliary pump lasers with respect to their respective resonance frequencies, and both normalized to half of the full-width at half-maximum (FWHM) of the resonance (Δ*ω*_0_). *β*_p,*n*_ are the *n*th-order dimensionless dispersion coefficients at the primary pump mode, normalized as *β*_p,*n*_ = −2*D*_p,*n*_/Δ*ω*_0_. *μ*_a_ is the mode number of the auxiliary mode relative to the primary pump mode with the value *μ*_a_ = 120. The LLE simulations are performed numerically using the split-step Fourier method. As in the experiments, the primary pump frequency is scanned from the blue- to the red-detuned side of its resonance until stable soliton states are generated. During frequency scanning of the primary pump laser, the auxiliary laser detuning (*α*_a_) is kept constant. As a result, the frequency difference between the two pumps is time-dependent. However, the two combs’ repetition rates synchronize irrespective of the two pump detunings (*α*_a_ and *α*_p_), as long as the primary microcomb is in a single- or multi-soliton state (see Note 2 in the Supplementary Information). In the simulations, the parameters are *β*_p,2_ = −0.2140, *β*_p,3_ = 0.0046, |*F*_a_|^2^ = 8, |*F*_p_|^2^ = 16. Calculated from these dispersion coefficients, the group velocity mismatch *γ* between the primary and auxiliary pump modes is −3.62, normalized to the FWHM as *γ* = (*D*_a,1_ − *D*_p,1_)/Δ*ω*_0_, meaning that *D*_p,1_ > *D*_a,1_. The negative sign of *γ* causes the P2 DW to appear on the red side of the auxiliary mode (see also next section for a discussion of the phase-matching conditions of the DWs).

For the case, in which the auxiliary laser is coupled into a mode that does not belong to the soliton mode family, we solve a system of two simultaneous generalized LLEs, which are expanded with XPM and a group velocity mismatch *γ* between the primary and auxiliary pump modes^25,26^:

4 $$\frac{{\partial \psi _{\mathrm{p}}\left( {\theta ,\tau } \right)}}{{\partial \tau }} = - (1 + i\alpha _{\mathrm{p}})\psi _{\mathrm{p}} + i\left( {\left| {\psi _{\mathrm{p}}} \right|^2 + \sigma \left| {\psi _{\mathrm{a}}} \right|^2} \right)\psi _{\mathrm{p}} - \mathop {\sum}\limits_{n = 2}^{N \ge 2} {( - i)^{n + 1}\frac{{\beta _{{\mathrm{p}},n}}}{{n!}}} \frac{{\partial ^n\psi _{\mathrm{p}}}}{{\partial \theta ^n}} + \gamma \frac{{\partial \psi _{\mathrm{p}}}}{{\partial \theta }} + F_{\mathrm{p}},$$

5 $$\frac{{\partial \psi _{\mathrm{a}}\left( {\theta ,\tau } \right)}}{{\partial \tau }} = - (1 + i\alpha _{\mathrm{a}})\psi _{\mathrm{a}} + i\left( {\left| {\psi _{\mathrm{a}}} \right|^2 + \sigma \left| {\psi _{\mathrm{p}}} \right|^2} \right)\psi _{\mathrm{a}} - \mathop {\sum}\limits_{n = 2}^{N \ge 2} {( - i)^{n + 1}\frac{{\beta _{{\mathrm{a}},n}}}{{n!}}} \frac{{\partial ^n\psi _{\mathrm{a}}}}{{\partial \theta ^n}} - \gamma \frac{{\partial \psi _{\mathrm{a}}}}{{\partial \theta }} + F_{\mathrm{a}},$$

where *ψ*_p_(*θ,τ*) and *ψ*_a_(*θ,τ*) are the intracavity primary and auxiliary field envelopes, respectively. *σ* is the XPM coefficient (2/3 for orthogonal polarization and 2 for the same polarization assuming perfect spatial mode overlap, otherwise less). Although *σ* is difficult to determine experimentally, varying it from 2/3 to 2 does not affect the simulation much, resulting mostly in slightly different amounts of redshift of the center frequency of the soliton comb envelope. *β*_a,*n*_ are the *n*th-order dimensionless dispersion coefficients, normalized as *β*_a,*n*_ = −2*D*_a,*n*_/Δ*ω*_0_. In the simulations, the parameters are *β*_p,2_ = −0.2140, *β*_p,3_ = 0.0046, *β*_a,2_ = 0.1441, *β*_a,3_ = 0.0031, |*F*_a_|^2^ = 8, |*F*_p_|^2^ = 16, *σ* = 1.5, and *γ* = 2.5. Here, the positive sign of *γ* determines the appearance of the DW on the blue side of the auxiliary mode as shown in Fig. 5c (see also discussion of phase-matching condition in next section). In addition, based on the same simulation parameters, a number of numerical simulations are carried out to determine how far the auxiliary pump can be away from the primary pump, while keeping the repetition rates of both combs synchronized. The simulation (Supplementary Fig. 3) shows that the two combs synchronize with each other in time domain, even with the auxiliary pump all the way out at 970.9 nm, where the normalized group velocity mismatch *γ* is around −103. However, as the group velocity mismatch increases, the power in the auxiliary comb modes is significantly reduced and the comb bandwidth becomes narrower.

### Phase-matching of the DW

A DW in the auxiliary frequency comb is expected to occur at a mode number *μ*′ at which the comb mode is exactly on resonance with the resonator mode. Thus *μ*′ satisfies the condition^36^

6 $$\omega _{\mathrm{a}} + \omega _{{\mathrm{rep}}}\mu ^{\prime} = \omega _{{\mathrm{a}},0} + D_{{\mathrm{a}},1}\mu ^{\prime} + D_{{\mathop{\rm{int}}} ,{\mathrm{a}}}\left( {\mu ^{\prime}} \right).$$

Here, *ω*_a_ is the auxiliary laser frequency and *ω*_rep_ is the repetition rate of the auxiliary frequency comb. *ω*_a,0_ is the auxiliary mode frequency, *D*_a,1_/2π is the FSR of the resonator at the auxiliary mode, and *D*_int,a_(*μ*′) is the integrated dispersion relative to the auxiliary pump mode. Equation (6) can be rearranged to

7 $$D_{{\mathop{\rm{int}}} ,{\mathrm{a}}}\left( {\mu ^{\prime}} \right) = \left( {\omega _{{\mathrm{rep}}} - D_{{\mathrm{a}},1}} \right)\mu ^{\prime} + \omega _{\mathrm{a}} - \omega _{{\mathrm{a}},0},$$

which is shown as the dashed line in Fig. 5f, g.

In the first case, in which the auxiliary laser is coupled into an optical mode of the primary soliton mode family and *D*_a,1_ = *ω*_rep_, the slope of the right-hand side of Eq. (7) with respect to the mode number *μ*′ is zero, inducing a DW at a mode number of around −100 (as shown in Fig. 5f), which is determined by the higher-order dispersion. However, as a result of XPM with the primary pump, the repetition rate (*ω*_rep_) of the auxiliary frequency comb is synchronized with that of the primary soliton, which mainly depends on *D*_p,1_. The slope of the right-hand side of Eq. (7) is positive, since *ω*_rep_ is larger than *D*_a,1_ (*γ* < 0). This can be interpreted by the dashed line plotted in Fig. 5f, inducing phase matching at a mode number of 61 (crossing point between dashed line and *D*_int,a_(*μ*), marked with P2), corresponding to the DW marked P2 in Fig. 5a.

For the second case, in which the auxiliary laser is coupled into a mode that is not part of the primary soliton mode family, similarly, due to the XPM-forced synchronization of the repetition rate, the slope of the right-hand side of Eq. (7) is negative due to *ω*_rep_ being smaller than *D*_a,1_ (*γ* > 0). This induces a phase-matching point at mode number 174 (crossing point between the dashed line and *D*_int,a_(*μ*) in Fig. 5g, marked with DW), on the blue side of the auxiliary pump, corresponding to the DW in Fig. 5c. Moreover, the wavelength of the DW can be controlled by simply placing the auxiliary pump at a different wavelength (Supplementary Fig. 4). Pumping at a different auxiliary wavelength will change the group velocity mismatch *γ* between the auxiliary pump and primary pump mode, and modifies the slope of the right-hand side of Eq. (7), which induces a different phase-matching condition for the DW.

## Supplementary information

Supplementary Information

## Data Availability

The data that support the findings of this study are available from the corresponding author upon reasonable request.

## Code availability

The code for the data analysis in this article is available from the corresponding author upon reasonable request.
