# Supplementary material for: Spectral extension and synchronization of microcombs in a single microresonator
Source: Nat Commun. 2020 Dec 14;11:6384. doi: 10.1038/s41467-020-19804-8 (PMC7736327; doi:10.1038/s41467-020-19804-8)
Supplement: Supplementary file 1 — Supplementary Information [file 41467_2020_19804_MOESM1_ESM.pdf]

Supplementary Information

Spectral Extension and Synchronization of Microcombs in a Single Microresonator

Shuangyou Zhang[1,2], Jonathan M. Silver[2,3], Toby Bi[1,4], and Pascal Del'Haye[1,2,4*]

[1]*Max Planck Institute for the Science of Light, 91058 Erlangen, Germany*

[2]*National Physical Laboratory (NPL), Teddington, TW11 0LW, United Kingdom*

[3]*City, University of London, London, EC1V 0HB, United Kingdom*

[4]*Department of Physics, Friedrich Alexander University Erlangen-Nuremberg, 91058 Erlangen, Germany*

**pascal.delhaye@mpl.mpg.de*

Supplementary Note 1: Optical spectrum of chaotic states

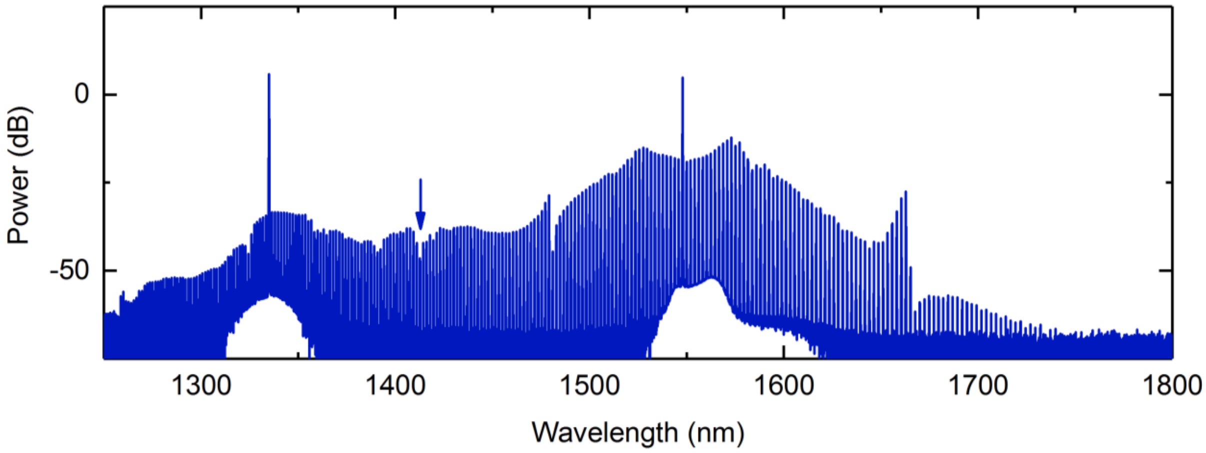

Supplementary Figure 1. Experimental optical spectrum of the chaotic states with the auxiliary laser pumping an optical mode (at 1335 nm) of the primary soliton's mode family. These optical spectra show an avoided mode crossing around 1410 nm but no dispersive wave around 1430 nm.

Supplementary Figure **1** shows the optical spectrum of the chaotic states with the auxiliary laser pumping an optical mode (~1335 nm) from the soliton mode family. The optical spectrum shows a very strong avoided mode crossing (AMC) around 1663 nm and a weak one around 1410 nm (marked with arrow). However, there is no dispersive wave around 1430 nm, which is different from the soliton spectra shown in Fig. 2 of the main text. Therefore in the simulation, as shown in Fig. 5f in the main text, only one AMC is added around 1410 nm (relative mode number 71 to the primary pump mode) without a phase-matching point for the DW around 1430 nm in the integrated dispersion D_{int}.

Supplementary Note 2: Comb repetition-rate synchronization at different pump detunings

Supplementary Figure **2** shows numerical simulation results for different detunings of the primary pump (α_p) and auxiliary pump laser (α_a). The remaining parameters (dispersion, pump modes and pump powers) are the same as these in Fig. 5c in the main text. Supplementary Figure **2a** shows the simulated intracavity optical spectrum when the primary soliton microcomb is in a single-soliton state and α_a = 2.6, α_p = 33. Supplementary Figure **2b** shows the simulated temporal waveforms of the primary single soliton (black, left axis) and the auxiliary optical pulse (red, right axis), corresponding to the spectrum shown in Supplementary Figure **2a**. Supplementary Figure **2c**,**d** show the simulated results with α_a = 2.6, α_p= 30. We also changed the auxiliary pump detuning in the simulation. The results are shown in Supplementary Figure **2e**,**f** with α_a = 2.8, α_p= 30. The repetition rate of two combs remains synchronized over a range of detunings, and therefore frequency differences, of the two pump lasers.

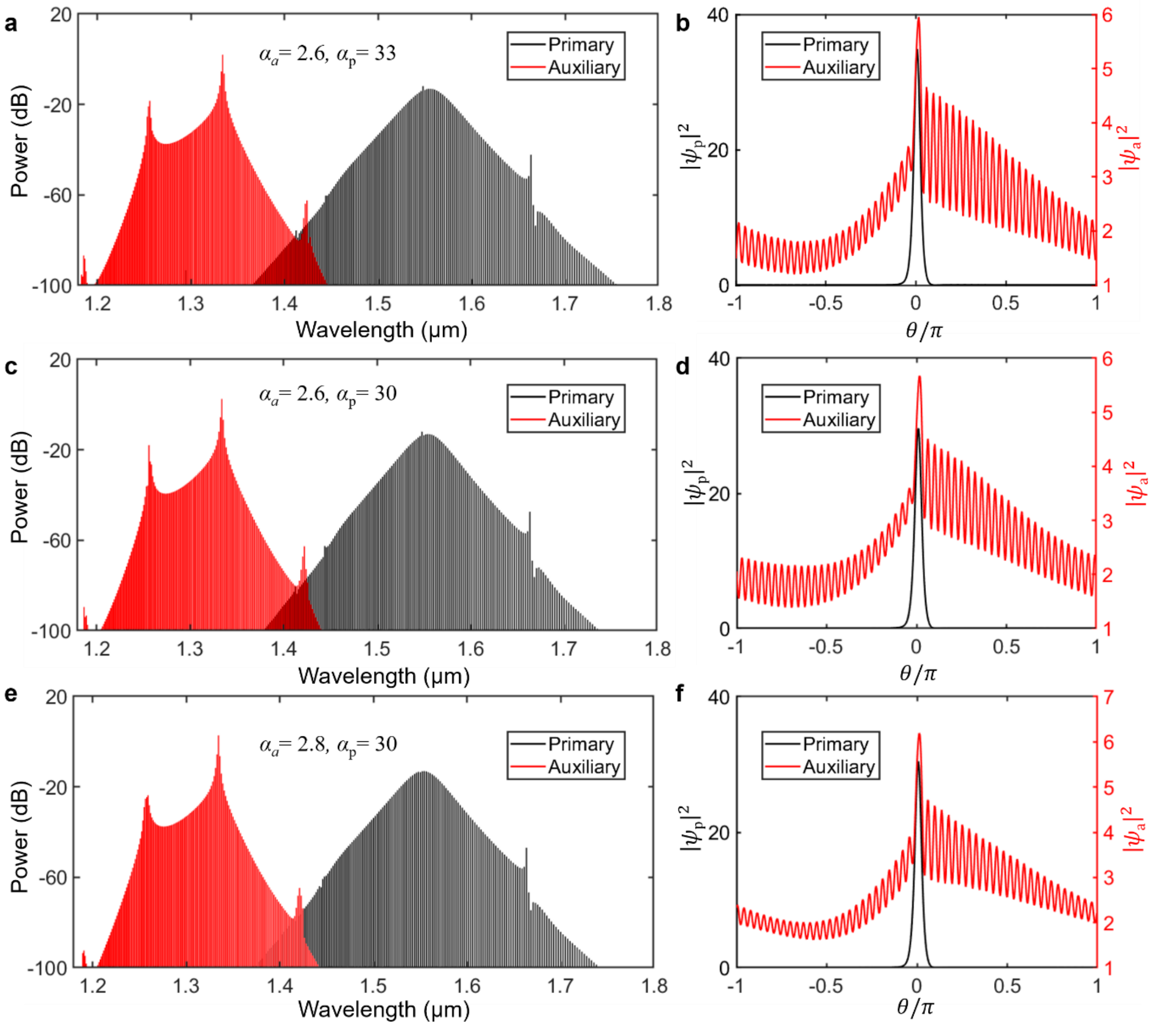

Supplementary Figure 2. Numerical simulation of intracavity optical spectra and temporal waveforms. Detunings of the auxiliary pump laser and primary pump laser are given by α_a and α_p, respectively. $|\psi_a|^2$ and $|\psi_p|^2$ are the corresponding intracavity powers.

Supplementary Note 3: Repetition-rate synchronization with the auxiliary pump laser at shorter wavelengths

Supplementary Figure **3** shows a simulation of a microcomb state with the auxiliary pump laser at 970.9 nm. The other simulation parameters (dispersion, pump modes and pump powers) are the same as the ones used in Fig. 5c of the main paper. The simulation result shows that the repetition rate of two combs can still synrchonize, however, the power and bandwidth of the auxiliary comb is significantly reduced.

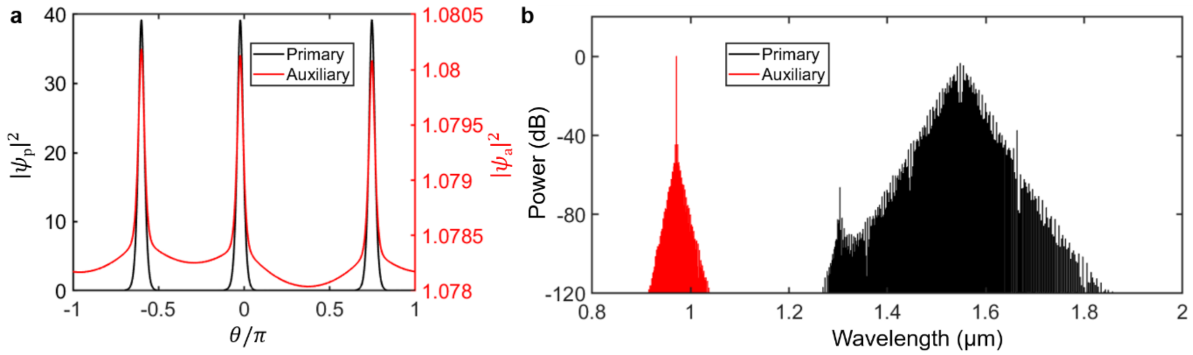

Supplementary Figure 3. Repetition-rate synchronization of microcombs with the auxiliary pump far away from the primary pump. a, Simulated temporal waveforms of the intracavity optical fields when the primary microcomb is in a 3-soliton state. $|\psi_a|^2$ and $|\psi_p|^2$ are the intracavity powers. **b**, Optical spectrum of the waveform shown in panel **a**. When the group velocity mismatch γ increases (due to the auxiliary pump being further away in frequency from the primary pump), the intensity of the auxiliary comb's pulses is reduced and the corresponding spectral bandwidth becomes narrower.

Supplementary Note 4: Control of the dispersive wave

Here, we show that the wavelength of the dispersive wave (DW) can be manually controlled by changing the wavelength of the auxiliary pump. Supplementary Figure **4** shows the simulated intracavity optical spectrum when placing the auxiliary pump laser at different wavelengths. Supplementary Figure **4a** shows the same results as plotted in Fig. 5c in the main paper, while Supplementary Figure **4b** shows a simulated optical spectrum when the auxiliary pump is at a wavelength of 1394 nm. We can see that, by changing the auxiliary pump wavelength (still the same auxiliary mode family as in Fig. 5c), the wavelength of the DW is significantly changed from 1255 nm to 1229 nm. When the auxiliary pump is at 1394 nm, the group velocity mismatch γ between the auxiliary pump and primary pump mode is $\gamma = 4.11$, compared to $\gamma = 2.5$ for the data in Supplementary Figure **4a**. This changes the phase-matching condition (crossing point between dash line and $D_{\mathrm{int,a}}(\mu)$ in Fig. 5g of the main paper) for the DW to a wavelength further away from the auxiliary pump.

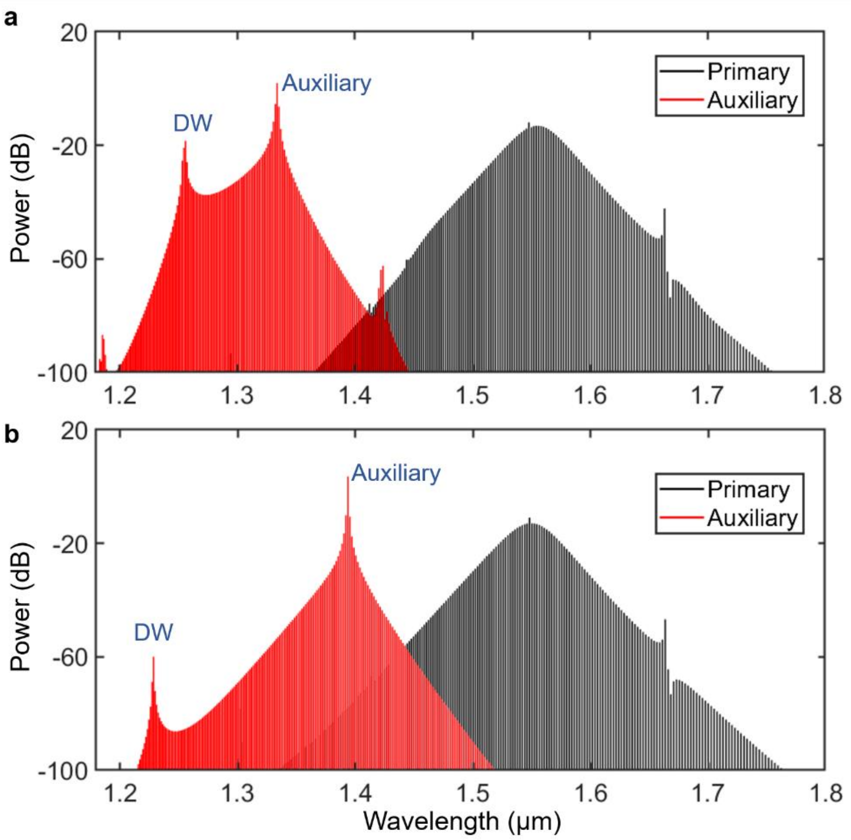

Supplementary Figure 4. Manual control of the DW wavelength. a, Simulated intracavity optical spectrum of a single-soliton primary microcomb with the auxiliary pump at 1334.6 nm and group velocity mismatch $\gamma = 2.5$. **b**, Simulated intracavity optical spectrum of a single-soliton primary microcomb with the auxiliary pump at 1394 nm and group velocity mismatch $\gamma = 4.11$. The wavelength of the DW changes from 1255 nm to 1229 nm when changing the auxiliary wavelength.

Supplementary Note 5: Spectral extension and synchronization of microcombs in a different microtoroid

To further confirm our findings, we performed similar experiments using a different microtoroid. Selecting an optical mode from the soliton mode family for the auxiliary pump, the extended optical spectrum of a single-soliton state microcomb is shown in Supplementary Figure **5a**. The inset is the optical spectrum in the region of overlap. In the same way as with Fig. 2 of the main text, only a single set of frequency comb modes is observed due to the limited resolution of the OSA. To detect the offset signal between the primary soliton microcomb and the auxiliary frequency comb, the primary soliton microcomb was operated in a multi-soliton state to increase the optical power in the region of overlap. Supplementary Figure **5b** is the corresponding RF spectrum of the offset signal. Indeed, a single RF peak at around 1.1 GHz is observed, further confirming that the repetition rates of the two combs are locked to one another and that this method could be used for the coherent extension of microcombs.

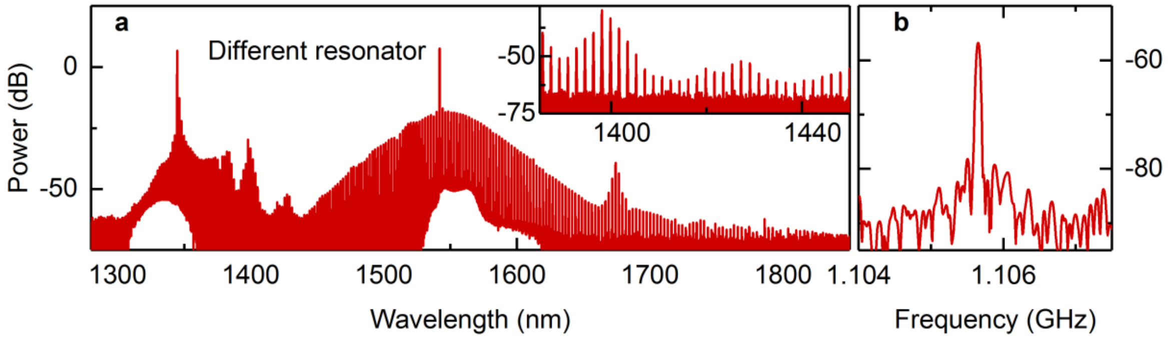

Supplementary Figure 5. Spectral extension and repetition-rate synchronization of microcombs in a different microtoroid. a, Optical spectrum of a single-soliton state generated using a different microtoroid with the primary and auxiliary pumps coupled into the same optical mode family. **b**, RF spectrum of the beat signal between the primary soliton microcomb and the auxiliary frequency comb with a 50 kHz resolution bandwidth, when the primary soliton microcomb is in a multi-soliton state.
